# Integrated lipidome and miRNome analyses reveal sex-based differences in circulating extracellular vesicles of alcohol use disorder patients

**DOI:** 10.1007/s10565-026-10213-z

**Published:** 2026-05-25

**Authors:** Carla Perpiñá-Clérigues, Susana Mellado, Cristina Galiana-Roselló, Saritha Kodikara, Blanca Martín-Urdiales, Miguel Marcos, Kim-Anh Lê Cao, Francisco García-García, María Pascual

**Affiliations:** 1 Department of Physiology, School of Medicine and Dentistry, University of Valencia, Avda. Blasco Ibáñez, 15, 46010 Valencia, Spain; 2 Computational Biomedicine Laboratory, Príncipe Felipe Research Center, C/Eduardo Primo Yúfera, 3, 46012 Valencia, Spain; 3 Melbourne Integrative Genomics, School of Mathematics and Statistics, University of Melbourne, Melbourne, VIC Australia; 4 Department of Internal Medicine, University Hospital of Salamanca, University of Salamanca, Institute of Biomedical Research of Salamanca, 37007 Salamanca, Spain

**Keywords:** Lipidomics, MiRNA transcriptomics, Multi-omics, Extracellular vesicles, Alcohol use disorder, Sex-based differences

## Abstract

Integrated multi-omics and extracellular vesicle (EV) analysis are emerging as powerful, complementary strategies for biomarker discovery. These approaches offer promising tools to enhance early detection, diagnosis, and treatment of alcohol use disorder (AUD). Here we applied an integrated miRNomic and lipidomic approach to analyze plasma EVs from AUD patients and controls of both sexes to gain a comprehensive understanding of the underlying molecular mechanisms. We identified an AUD signature with predictive potential for diagnostic applications, representing an initial step in this direction. Individual features (e.g., hsa-miR-99b-3p, hsa-miR-556-5p, Cer_NDS-d39:1, and PI18:0_18:2) represented important components; however, the strength of this signature lay in the combined profile rather than isolated markers. We also revealed an AUD-sex signature that provided insight into how biological responses to alcohol differ between females and males (including features such as hsa-miR-1301-3p and PC39:4), which also underscored the power of multi-omic integration. The individual miRNome approach also revealed an opposite functional alteration by sex in various alcohol related systems, such as pathways associated with immunity, oxidative stress, and autophagy. An open-access Shiny web application (https://carpercle.shinyapps.io/SexEVEthOmics/) accompanies this study, providing interactive access to the complete dataset and additional analyses for customized exploration. Together, our findings underscore the added value of multi-omics integration in identifying disease-associated molecular signatures. This sex-informed approach highlights a promising avenue for the development of more personalized diagnostic tools and therapeutic strategies in AUD.

**Graphical Abstract:**

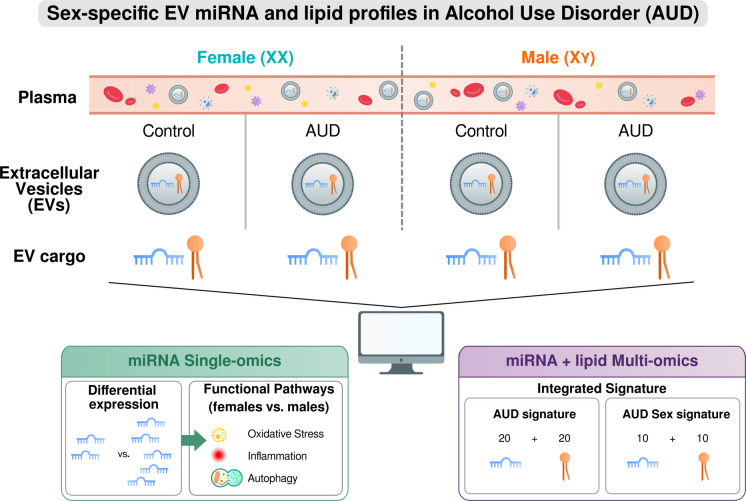

**Supplementary Information:**

The online version contains supplementary material available at 10.1007/s10565-026-10213-z.

## Introduction

Multi-omics has emerged as a result of advances in various omic-based technologies (including genomics and metabolomics) alongside enhanced computing capabilities, enabling a better understanding of the complex molecular interplay between health and disease by combining the power of individual data types (Babu and Snyder 2023). This analytical approach measures different layers of cellular information, facilitating the identification of molecular signatures and biomarkers for mechanism-based classifications and tailored therapeutic interventions (Ma et al. 2021; Tarazona et al. 2021) and offering deep insight into the pathophysiology of disease states (Maan et al. 2023). Single-omic studies commonly apply univariate approaches, such as differential expression or regression analyses, to identify individual biomarkers, whereas multi-omics studies use multivariate integration methods, including PLS-based approaches, matrix factorization techniques, and machine learning strategies, to investigate correlations and coordinated patterns across multiple molecular layers (Rohart et al. 2017). These integrative strategies may improve disease prediction, prevention, and treatment by considering each individual's genetics, environment, and lifestyle factors (Babu and Snyder 2023).

Recent reports support the applicability of transcriptomics and lipidomics in biomarker identification and pathology elucidation. Transcriptomics, including miRNA analysis, represents a robust methodology for determining mRNA/miRNA expression via high-throughput RNA-sequencing (RNA-seq) (Maan et al. 2023). Although lipidomics represents an effective approach to analyzing changes to the levels of endogenous lipids in biological systems (Maan et al. 2023), alterations in lipid levels/enzymatic reactions may not accurately identify pathways altered in specific pathologies. We can address this gap by integrating various omic-based technologies, which offer a more comprehensive view of alterations in complex biological processes induced by any pathological condition. A combined miRNomic and lipidomic approach has the potential to generate extensive datasets that will support the identification of associations between miRNAs and lipids/enzymes and uncover molecular mechanisms based on high-throughput data.

An increasing number of studies into alcohol consumption have applied transcriptomic and lipidomic technologies to understand the molecular mechanisms underlying alcohol use disorders (AUDs) and search for possible disease biomarkers (Hitzemann et al. 2022; Lim et al. 2021; Perpiñá-Clérigues et al. 2024). Alcohol remains a widely misused substance, with the World Health Organization reporting that abuse causes approximately 2.6 million deaths per year (Geneva: World Health Organization 2024). Early AUD identification and treatment potentially reduce the risk of developing related pathologies (e.g., certain types of cancer, diseases associated with reduced immunity, and increased infections, infertility, and depression) (Mostafa et al. 2017). While traditional clinical biomarkers such as AST, ALT, or circulating DNA are useful for assessing alcohol-related organ damage, they do not capture the complex neurobiological underpinnings of AUD itself, nor do they predict disease progression or treatment response (Clark et al. 2025). Overall, the lack of specific and sensitive biomarkers for AUD diagnosis and effective treatment approaches has prompted the need for the application of novel technologies such as integrated multi-omics. Multi-omics enables the simultaneous analysis of miRNAs and lipids, for example, to provide insights into miRNomic and lipidomic alterations following alcohol consumption, which will help to map complex relationships.

Extracellular vesicles (EVs), which carry miRNAs and lipids, act as critical intercellular mediators, particularly under cellular stress or damage. Unlike synthetic drug delivery systems, EVs demonstrate superior biocompatibility, extended biodistribution, and low immunogenicity (Clayton et al. 2003). These properties position EVs and their content as valuable non-invasive biomarkers and therapeutic targets for diverse pathologies, including neurodegenerative disorders (Donoso‐Quezada et al. 2021; Su et al. 2021; Yuan et al. 2022). Notably, evidence indicates that alcohol exposure significantly alters EV biology, including their biogenesis, release, and molecular cargo composition (Ibáñez et al. 2020, 2019). In different cellular contexts, ethanol has been shown to increase EV secretion and modulate the enrichment of specific miRNAs and inflammatory mediators, thereby influencing intercellular communication and contributing to alcohol-induced tissue damage and neuroinflammation. We previously identified AUD-related lipidomic fingerprints in plasma EVs from AUD females and males (Perpiñá-Clérigues et al. 2024); here, the first implementation of an integrated multi-omics approach (combining transcriptomics and lipidomics) using plasma EVs isolated from AUD males and females has enabled a more comprehensive understanding of the biological processes involved following excessive alcohol consumption.

## Materials and methods

### Human subjects and experimental design

This study includes AUD patients (according to DSM-5 criteria) referred to the Alcoholism Unit of the University Hospital of Salamanca (Spain) (Fernández-Regueras et al. 2023). All patients in this group actively drank ≥ 90 g of ethanol/day until entering the study. Healthy volunteers who reported drinking < 15 g of ethanol/day were also recruited as control patients. AUD patients and healthy volunteers were previously recruited and described in Perpiñá-Clérigues et al. 2024. The total cohort comprised 25 individuals: 12 AUD (5 females, 7 males) and 13 controls (7 females, 6 males). The same individuals were used across lipidomics, miRNA transcriptomics, and multi-omic integration; however, due to sample availability and quality control filtering, the number of samples varied by omic (see Table S1 for details). All individuals gave their informed consent to participate, and the study was approved by the Ethics Committee of the University Hospital of Salamanca (Spain). Plasma samples were snap-frozen in liquid nitrogen and stored at −80 °C until further use. At this point, samples were processed for EV isolation.

**Fig. 1:**
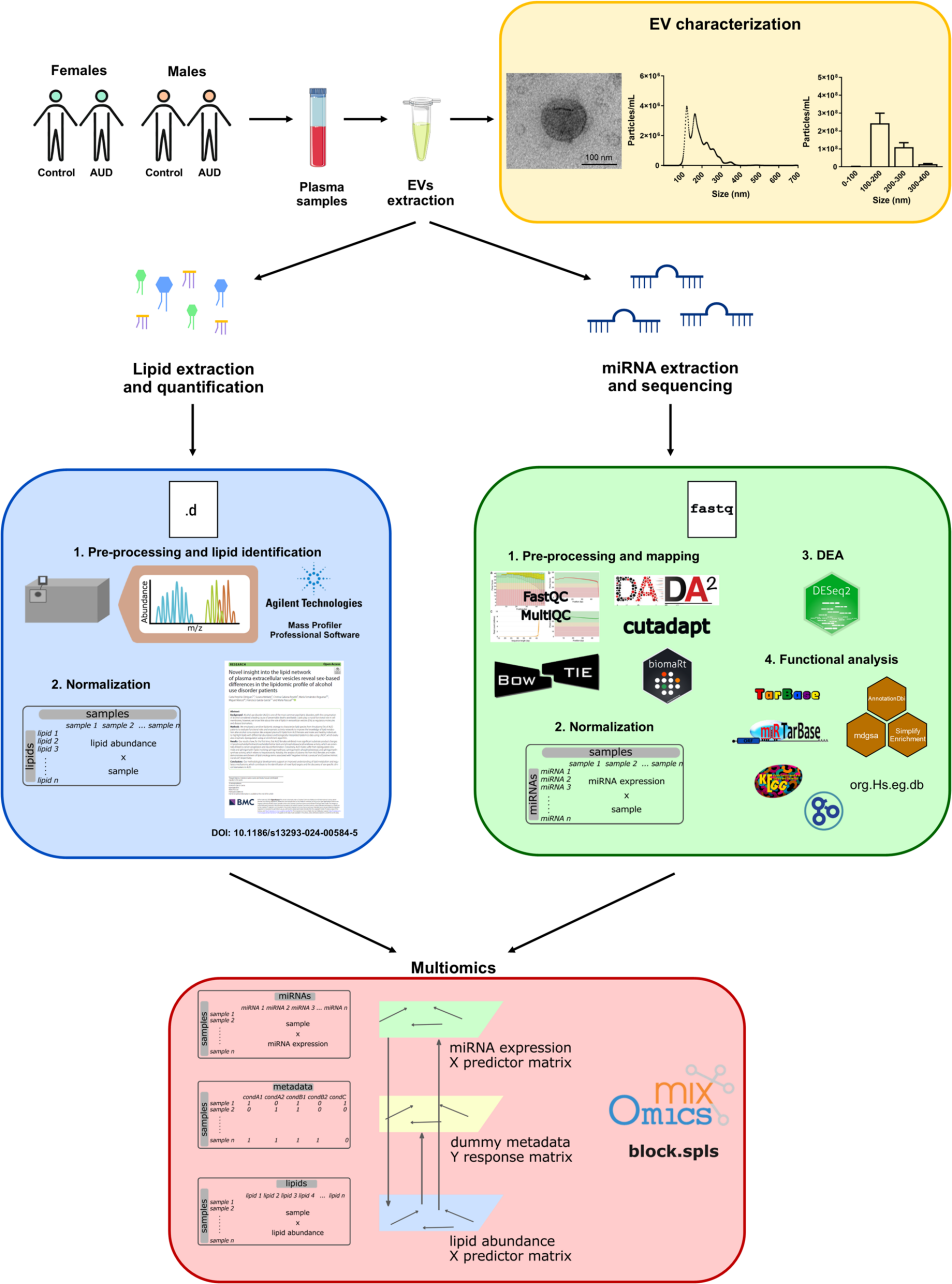
**Experimental design and workflow.** Lipids and miRNAs were extracted from plasma extracellular vesicles (EVs) of alcohol use disorder (AUD) patients and healthy individuals of both sexes. The lipidome and miRNome were analyzed independently before the data were integrated to examine reciprocal regulation. The lipidome analysis was previously conducted by Perpiñá-Clérigues et al. 2024, who detailed the workflow from lipid extraction to bioinformatics characterization of EV lipid content across experimental groups. The miRNome analysis involved four key steps: (**i**) Extraction and sequencing of EV miRNAs; (**ii**) Preprocessing, mapping, and normalization; (**iii**) Differential expression analysis; and (**iv**) Functional profiling. The integration of miRNomic and lipidomic datasets begins with the normalization of expression matrices. The implementation of the mixOmics R package applied the block sparse partial least squares (sPLS) method for multi-omics integration. Detailed sample correspondence across omics is provided in Table S1

Investigating the effects of alcohol consumption on miRNA and lipid content in EVs and exploring the molecular basis underlying sex-specific responses used the strategy depicted in Fig. 1. Lipids and miRNAs were obtained from plasma EVs of AUD and control patients.

### EV Isolation and characterization

Plasma EVs were isolated from 250 μL of human plasma using the Total Exosome Isolation Kit (Invitrogen, USA; Cat. #4484450) and characterized via nanoparticle tracking analysis (NTA) using a NanoSight NS300 (Malvern Panalytical, UK) to determine size distribution and concentration. EV morphology was confirmed by transmission electron microscopy (TEM) using a FEI Tecnai G2 Spirit (FEI Europe, Netherlands), showing characteristic vesicular structures. EV validation is presented in the Supplementary Material (Fig. 1 and S1).

### RNA extraction and sequencing

Total RNA from plasma EVs was isolated using the Total Exosome RNA Isolation Kit (Invitrogen, Waltham, MA, USA), following the manufacturer's instructions. The construction of miRNA libraries was performed using the TruSeq Small RNA Library Preparation Kit from Illumina (San Diego, CA, USA) at the Genomics and Genetics Service of the Príncipe Felipe Research Center (Valencia, Spain). RNA transcripts were first converted into complementary (c)DNA libraries, followed by quality and quantity assessments employing High Sensitivity D1000 ScreenTape for TapeStation System (Agilent Technologies, Santa Clara, CA, USA). The final libraries were pooled in equimolar concentration for sequencing in the MiSeq™ System (Illumina) through MiSeq™ Reagent Nano Kit v2 (Illumina) with 1 × 50 bp read length. Next-generation sequencing was performed at the Centre for Genomic Regulation (CRG) in Barcelona (Spain). The raw data generated by the high-throughput sequencing of miRNA were exported as fastq files.

To validate the miRNA levels from plasma EVs, real-time quantitative PCR (RT-qPCR) was performed as previously described (Ibáñez et al. 2019). Details of the nucleotide sequences of the used miRNA assays can be found in the Supplementary Material (Table S2).

### Processing and mapping

Raw sequencing reads underwent preprocessing using Cutadapt (v.4.6) (Martin 2011). Sequences between 17–35 nucleotides and with Phred scores ≥ 30 were retained (-a TGGAATTCTCGGGTGCCAAGG -m 17 -l 35 -q 30). Illumina adapters were removed before downstream analysis. Trimmed reads were mapped to human mature miRNAs from miRBase (mature.fa) (Griffiths-Jones 2006) using Bowtie2 (v2.5.3) (Langmead and Salzberg 2012). The alignment parameters (-L 6 -i S,0,0.5 –ignore-quals –norc –score-min L,−1,−0.6 -D 20 –very-sensitive) were adapted from Locati e*t al.* (Locati et al. 2015), optimizing sensitivity and specificity. Read counts were extracted from the SAM files, and only uniquely mapped reads—filtered via Bash—were used for quantification to avoid biases from multimapping.

### Exploratory analysis and normalization of miRNA profiles

Custom R scripts utilizing DESeq2 (v.1.42.1) (Love et al. 2014) were used for both filtering and normalization. miRNAs ≥ 10 reads in at least three samples were retained. Subsequently, the median ratio normalization method implemented in DESeq2 was applied to adjust for differences in sequencing depth and RNA composition. Boxplots, PCA, and clustering—performed pre- and post-normalization—assessed group distribution, batch effects, and data quality. All statistical analyses and data visualization scripts were executed in R (v4.3.2) (R Development Core Team, n.d.) using RStudio as the integrated development environment.

### Differential expression analysis of miRNAs

Identification of differentially expressed miRNAs was performed using DESeq2 (Love et al. 2014). P-values were adjusted for multiple testing using the Benjamini–Hochberg (BH) method (Benjamini and Hochberg 1995), with statistically significant miRNAs defined as those with a BH-adjusted p-value ≤ 0.05. Log2 fold change shrinkage was applied using the ashr method to improve the estimation of effect sizes.

Three primary comparisons were performed to assess sex-specific effects of AUD:

1. AUD Impact in Females (**IF**) assesses the differences between AUD females and control females (AUD.Females—Control.Females)
2. AUD Impact in Males (**IM**) assesses the differences between AUD males and control males (AUD.Males—Control.Males)
3. Impact of Sex in AUD (**IS**) assesses the differences between IF and IM (AUD.Females—Control.Females)—(AUD.Males—Control.Males)

Expression changes were quantified using log fold change (LFC), where the absolute value represents the magnitude of change and the sign indicates direction. A positive LFC indicates higher expression in the first group (AUD groups for IF/IM; females for IS), while a negative LFC indicates higher expression in the second group (controls for IF/IM; males for IS). For the IS comparison, which examines sex-dependent responses to AUD, the interpretation requires careful consideration. To resolve these scenarios, individual miRNA trends in IF and IM must be cross-referenced (Figure S2, "*Help*" section of our website [https://carpercle.shinyapps.io/SexEVEthOmics/]).

A complementary analysis examined combined AUD versus control samples (without sex stratification) to assess whether sex-specific analysis provided additional insights (see Supplementary Material).

### Functional profiling of miRNAs

Differentially expressed miRNAs were linked to their experimentally validated target genes using combined data from TarBase (Skoufos et al. 2024) and miRTarBase (Cui et al. 2025). Next, their LFC and p-values were transferred to target genes using the mdgsa package (v.0.99.2) (Montaner and Dopazo 2010), reflecting both statistical significance and direction of regulation. This allowed modeling of the cumulative regulatory effect of multiple miRNAs on individual genes. Functional enrichment analysis of Gene Ontology (GO) terms (27) and Kyoto Encyclopedia of Genes and Genomes (KEGG) pathways (28) was performed using gene annotations from org.Hs.eg.db R package (v. 3.18.0) (Carlson 2017), with significance determined by BH-adjusted *p*-value ≤ 0.05. Pathways with divergent regulatory impacts between sexes were identified based on opposing log odds ratios (LORs), where the sign and magnitude of the LOR reflected the direction and strength of the enrichment effect. Visualization was carried out using simplifyEnrichment (Gu and Hübschmann 2023) (semantic similarity threshold = 0.8) and dot plots to illustrate functional divergence across conditions.

### Multi-omic integration

Data were analyzed using processed and normalized results from previous single-omics analyses. Only paired samples were included in the integration, as sample availability and quality control filtering caused the number of samples to vary by omic type (see Table S1 for details). To assess concordance between datasets, an initial sPLS analysis was performed on miRNA and lipid data, evaluating their alignment with AUD and sex conditions. Then, a multi-omic integration was then conducted using the N-integration approach via the block.sPLS method (mixOmics v.6.26.0) (Rohart et al. 2017), with miRNA and lipid levels as predictors (X) and a dummy matrix encoding AUD-sex groups as response (Y). The model was fitted using three components with a fully connected design between blocks (design = "full"). Feature selection retained 20, 20, and 10 features in X, and 6, 6, and 4 in Y for components 1 to 3, respectively. Variable contributions and inter-dataset relationships were explored using plotVar and plotLoadings.

### Web platform—SexEVEthOmics

In addition to the results presented in this article, an open-access, interactive web platform (https://carpercle.shinyapps.io/SexEVEthOmics/) was developed using the R shiny framework (v.1.10) (Chang et al. 2012). This tool enables users to access the full dataset and interactively explore differential miRNA expression results, functional analyses, lipidomic profiles, and multi-omics integration. The platform is structured into six sections and provides access to additional analyses and visualizations not included in the main manuscript. Some modules allow users to adjust parameters, offering opportunities for customized exploration and hypothesis generation.

## Results

### Sex-based differences in the miRNA expression profile of AUD patient plasma EVs

**Fig. 2:**
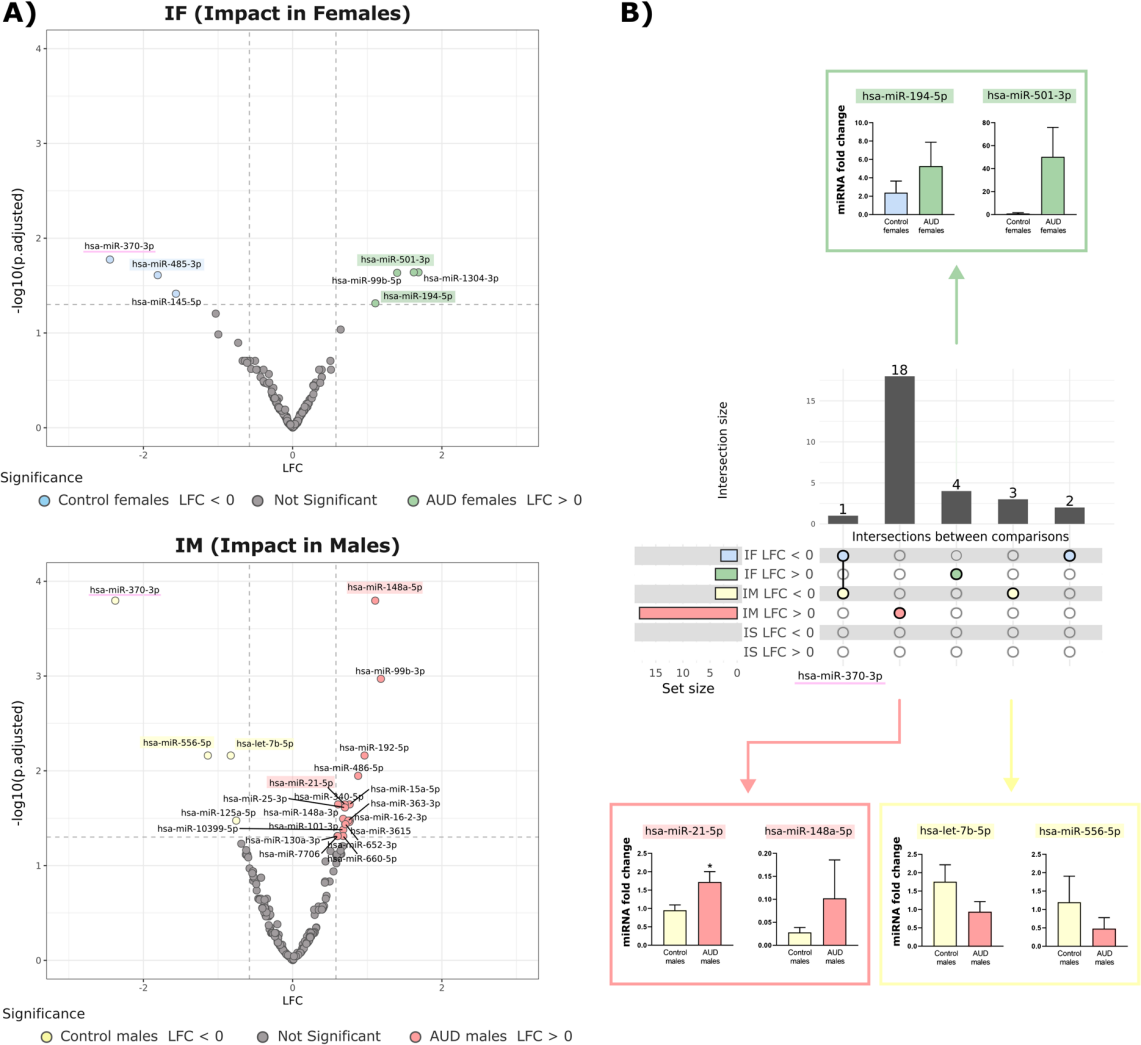
**Differential expression and validation of miRNAs in plasma EVs from AUD and control patients stratified by Sex.** **A**) Volcano plots showing differential miRNA expression (adjusted *p*-value < 0.05) in the IF and IM comparisons. Horizontal dashed lines indicate the adjusted *p*-value threshold (adjusted *p*-value = 0.05), and vertical dashed lines indicate the fold-change threshold (|LFC| = 0.58, corresponding to a 1.5-fold change). miRNAs with a positive log fold change (LFC > 0) display upregulation (green and red dots for IF and IM, respectively) and miRNAs with a negative LFC (LFC < 0) display downregulation (blue and yellow dots for IF and IM, respectively) in AUD patients. Highlighted miRNAs were validated by RT-qPCR, and underlined miRNA (hsa-miR-370-3p) represents a commonly significantly altered miRNA between sexes. **B**) Intersection plot displaying shared and unique significantly altered miRNAs across IF, IM, and IS comparisons and RT-qPCR validation of selected miRNAs: *hsa-miR-194-5p*, *hsa-let-7b-5p*, *hsa-miR-21-5p, hsa-miR-148a-5p, hsa-miR-556-5p, and hsa-miR-501-3p*. Data represent mean ± SEM, *n* = 4–6 samples/group. * *p* < 0.05, compared with healthy controls. One-way ANOVA with Tukey multiple comparisons testing was performed. Underlined miRNA (hsa-miR-370-3p) represents common significantly altered miRNA between sexes. *AUD: alcohol use disorder, IF: AUD Impact in Females, IM: AUD Impact in Males, IS: Impact of Sex in AUD.* AUD: *n* = 9 (3 females/6 males). Controls: *n* = 11 (6 females/5 males)

To implement our knowledge in the search for biomarkers in plasma EVs from AUD patients, we analyzed plasma EV samples from female and male AUD and healthy patients (controls) who had been previously assessed via lipidomics (Perpiñá-Clérigues et al. 2024). We investigated the role of miRNAs in the four experimental groups, defined by sex and chronic alcohol consumption. Figure 2A-B reports the upregulated (LFC > 0) and downregulated (LFC < 0) miRNAs. We observed 4 upregulated and 3 downregulated miRNAs in females and 18 upregulated and 4 downregulated miRNAs in males. Notably, the comparison in males revealed a higher number of upregulated miRNAs compared to females (Fig. 2A-B), suggesting distinct sex-specific responses to alcohol consumption. Notably, we failed to detect any significantly altered miRNAs in the IS comparison (Web Platform—Study Overview [https://carpercle.shinyapps.io/SexEVEthOmics/], Table S3).

We next validated six miRNAs identified from the miRNA-seq data using RT-qPCR (Fig. 2B). We selected several miRNAs (hsa-miR-194-5p, hsa-let-7b-5p, and hsa-miR-21-5p) involved in the immune response (Ibáñez et al. 2020; Lewohl et al. 2011; Ureña-Peralta et al. 2018; Yakovlev et al. 2024), which revealed significant changes in AUD patients. Consistent with the miRNA-seq results, hsa-miR-21-5p significantly increased, and hsa-miR-148a-5p tended to increase, in AUD males compared to healthy males, while hsa-let-7b-5p and hsa-miR-556-5p tended to decrease. In females, hsa-miR-194-5p and hsa-miR-501-3p tended to increase in AUD females compared to healthy females. Overall, these results suggest that these miRNAs may participate in AUD pathogenesis in a sex-dependent manner.

### Sex-based differences in miRNA functional analysis of plasma EVs from AUD patients

**Fig. 3:**
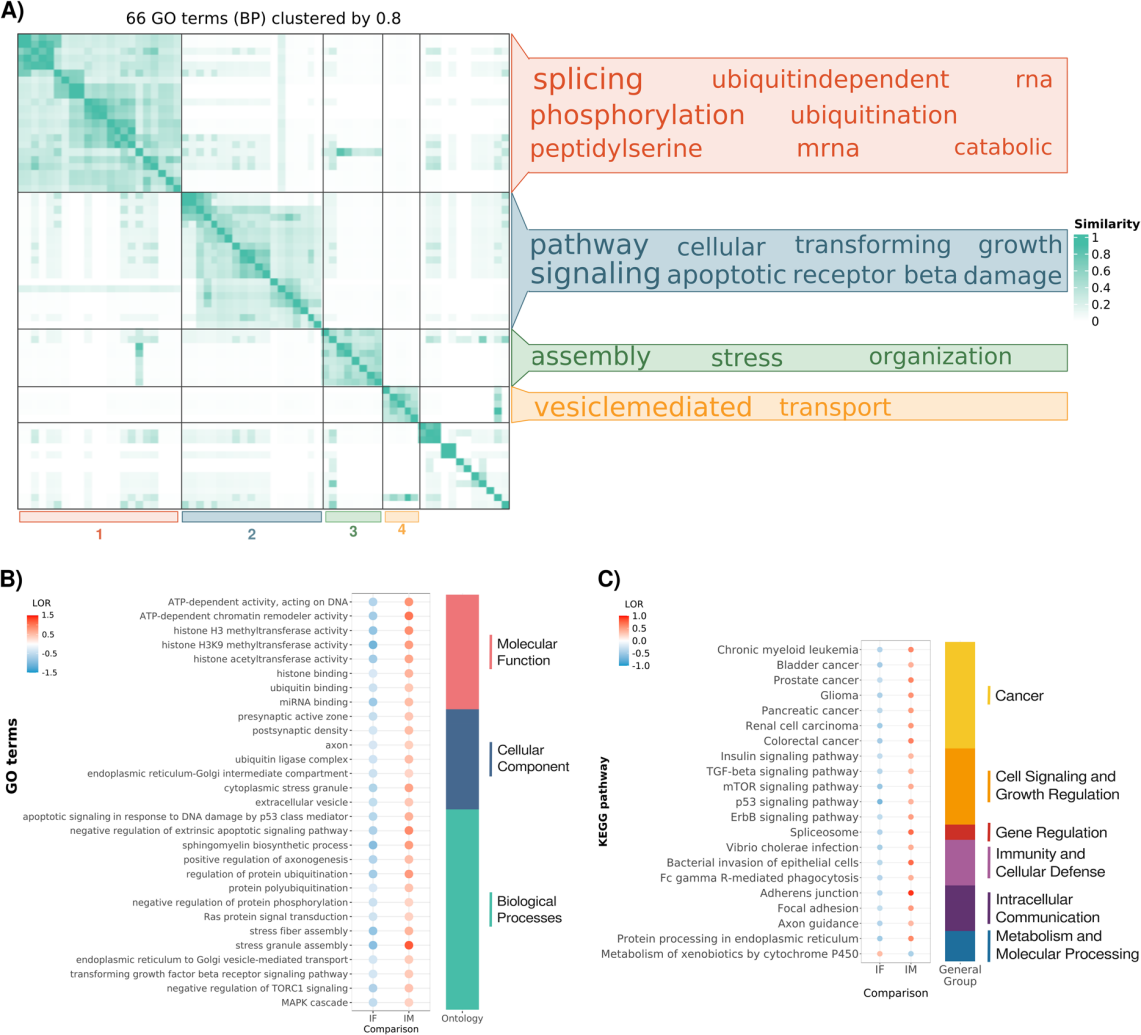
**Sex-specific functional signatures of plasma EV miRNAs in AUD patients—gene set analysis results.** **A**) Clustering of significant BP GO terms common across IF, IM, and IS comparisons based on semantic similarity (threshold ≥ 0.8). Word clouds summarize representative terms for each cluster. **B**) Dot plot depicting selected significant GO terms (absolute LOR > 0.3) across MF, CC, and BP ontologies. Dot color represents the LOR value, and the right-side color bars indicate the ontology category of each term. **C**) Dot plot illustrating significant KEGG pathways (absolute LOR > 0.3). Dot color represents the LOR value, while the right-side color bars classify pathways into general functional groups. *AUD: alcohol use disorder, BP: Biological Processes, CC: Cellular components, IF: AUD Impact in Females, IM: AUD Impact in Males, GO: Gene Ontology, KEGG: Kyoto Encyclopedia of Genes and Genomes, LOR: log odds ratio, MF: Molecular Functions*

We next assessed the functional impact of miRNA deregulation in AUD patient plasma EVs to provide biological insights into associated etiopathogenic pathways (Pers 2016). We performed gene set analysis (GSA) on validated target genes associated with the differentially expressed miRNAs, incorporating GO terms related to Biological Processes (BP), Cellular Components (CC), and Molecular Functions (MF), and KEGG pathways. Notably, we observed several significantly impacted GO terms and KEGG pathways across IF, IM, and IS comparisons, highlighting notable sex-based differences. Table S4 summarizes the number of significant GSA results across the IF, IM, and IS comparisons, while Figures S3–S6 display the common significant results between IF and IM for the BP, CC, and MF ontologies. Figure 3A presents clusters derived from 66 common BP GO terms that exhibit significant sex-specific variation across all comparisons, characterized by negative LOR values in females and positive LOR values in males. These processes cluster into four main functional groups: (1) gene expression and protein turnover, encompassing ubiquitin-dependent degradation, mRNA regulation, splicing, phosphorylation, and catabolic pathways; (2) cell signaling and fate determination, such as cellular transformation, apoptosis, growth regulation, and receptor-mediated signaling; (3) cellular structure and stress response; and (4) vesicle-mediated transport related to intracellular trafficking and membrane dynamics.

Figure 3B illustrates a selection of the most relevant significantly altered GO terms characterized by distinct sex-dependent patterns. The MF ontology highlighted terms related to histone modifications, including histone H3 methylation and ubiquitin-related processes. The CC ontology revealed an enrichment in terms related to synaptic activity, including the pre- and post-synaptic regions, the axon, and EVs. Within the BP ontology, significant terms associated with neurological functions (e.g., axonogenesis and lipid metabolism), cellular communication, and vesicle-mediated transport, including Golgi-related pathways, Target of Rapamycin Complex 1 (TORC1) signaling, protein phosphorylation, and Mitogen-Activated Protein Kinase (MAPK) cascades. Interestingly, only terms associated with DNA replication—specifically, the BP term "regulation of the DNA damage checkpoint" and the CC term "CMG complex"—exhibited a consistent pattern across IF and IM comparisons (Figures S3–S4).

Finally, KEGG pathway enrichment analysis revealed divergence between sexes in the functional impact of miRNA deregulation in AUD (Fig. 3C). In AUD females (IF), several pathways displayed a negative LOR, whereas these pathways possessed a positive LOR in AUD males (IM). These pathways participate in a range of disease-related processes, including cancer-associated pathways involving key genes (e.g., MAP2K1, BRAF, KRAS, MAPK3, ARAF, RAF1, and MAPK1), pathways related to cell signaling and growth regulation (e.g., mammalian target of rapamycin [mTOR], p53, and the family of receptor tyrosine kinases ErbB), immunity-related pathways, intracellular communication (e.g., axon guidance and adherens junctions), and metabolism and molecular processing associated with the endoplasmic reticulum.

### Integrated transcriptomic and lipidomic analyses reveal sex-based differences in AUD patient plasma EVs

We employed our miRNomic data, combined with lipidomics data from (Perpiñá-Clérigues et al. 2024), to explore novel relationships between miRNAs and lipids in plasma EVs isolated from AUD females and males. After data preprocessing and normalization according to their respective omic natures, we obtained a total of 387 miRNAs and 575 lipids for the same samples. Figure S7 illustrates the sPLS results, where the samples exhibit comparable spatial organization in both datasets, with high correlation values observed for the first two components (Comp1: 0.951 and Comp2: 0.953). This strong alignment suggests the successful application of the N-integration methodology (block.sPLS).

### Biological patterns captured by components 1 and 2: links to alcohol consumption and sex

**Fig. 4:**
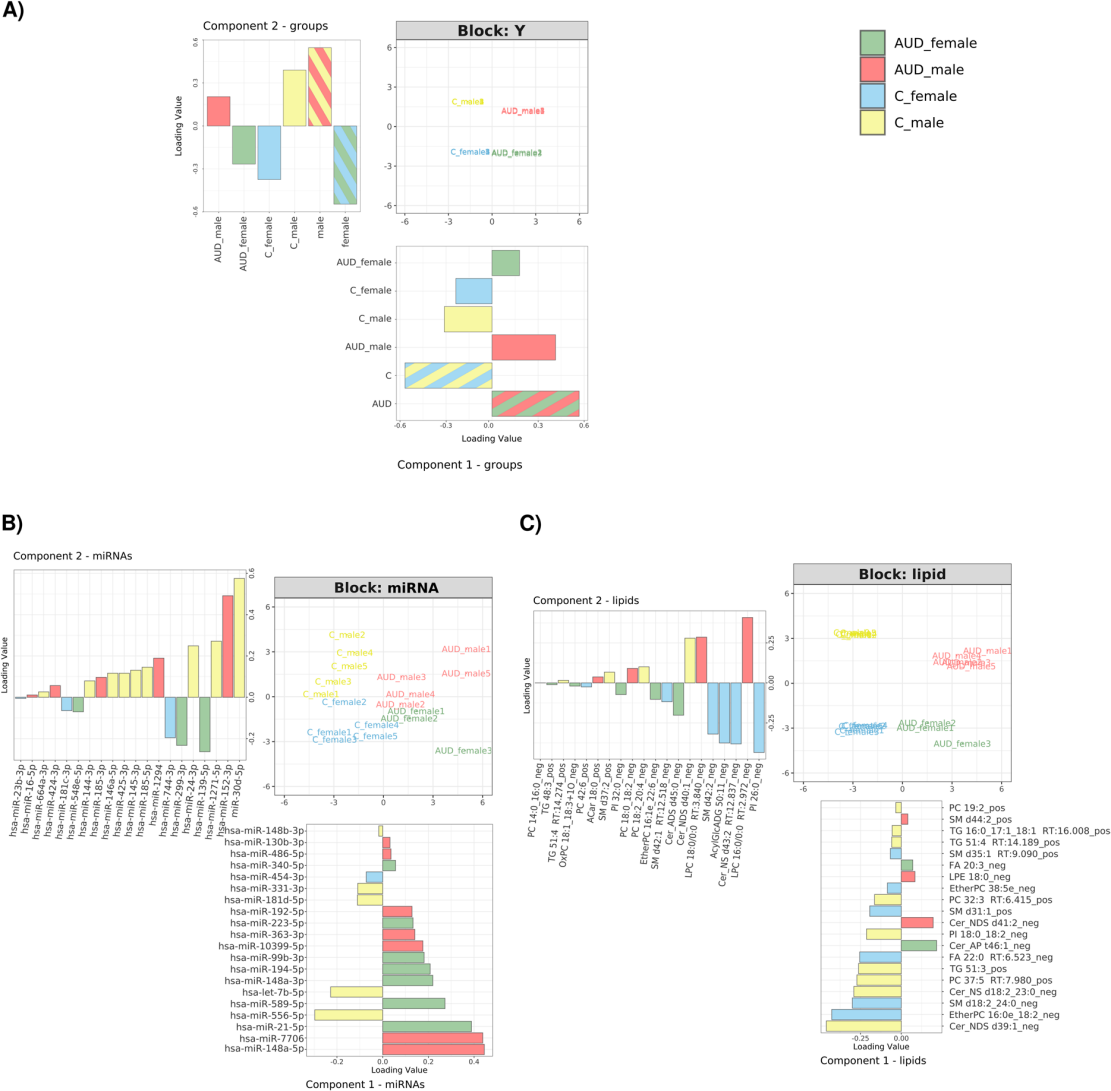
**AUD- and sex-driven sample separation based on integrated patient EV miRNA and lipid profiles. **Sample and loading plots from the block.sPLS integration analysis performed using mixOmics, representing Components 1 and 2. Samples are projected into the space defined by the components derived from the (**A**) metadata (Y), (**B**) miRNA, and (**C**) lipid dataset. The loading plots identify the most relevant variables contributing to each component: (**A**) metadata (Y), (**B**) miRNA, and (**C**) lipids. Component 1 is represented on the X-axis, with its corresponding loading plot displayed behind the sample plot. Component 2 is shown on the Y-axis, with its loading plot displayed to the left. Colors in the sample plots indicate the experimental groups. Colors in the loading plots denote which experimental group has the highest mean expression/abundance of that feature. AUD: *n* = 8 (3 females/5 males). Controls: *n* = 10 (5 females/5 males)

Performing dimension reduction using the mixOmics package produces latent components defined by their loading vectors, which indicate the weight of each original variable's contribution to the component. Figure 4 depicts the sample plot and the loadings plot for Components 1 and 2. Component 1 captures variance related to alcohol consumption, while Component 2 captures variance related to sex. The bar plots display the loading values, indicating the extent to which each feature contributes to the differentiation of the groups; those at the bottom and those with longer bars have the most significant influence. As a result, we identified a subset of 40 miRNAs (Fig. 4B) and 40 lipids (Fig. 4C) that may distinguish and clarify the influence of alcohol (e.g., hsa-miR-148a-5p, hsa-miR-7706, and hsa-miR-21-5p and Cer_NDS d39:1_neg, EtherPC 16:0e_18:2_neg, and SM d18:2_23:0) and sex (e.g., hsa-miR-30d-5p, hsa-miR-152-3p, and hsa-miR-1271-5p and Cer_NDS d39:1_neg, EtherPC 16:0e_18:2_neg, and SM d18:2_23:0). Table S5 details the description of lipid subclass abbreviations.

**Fig. 5:**
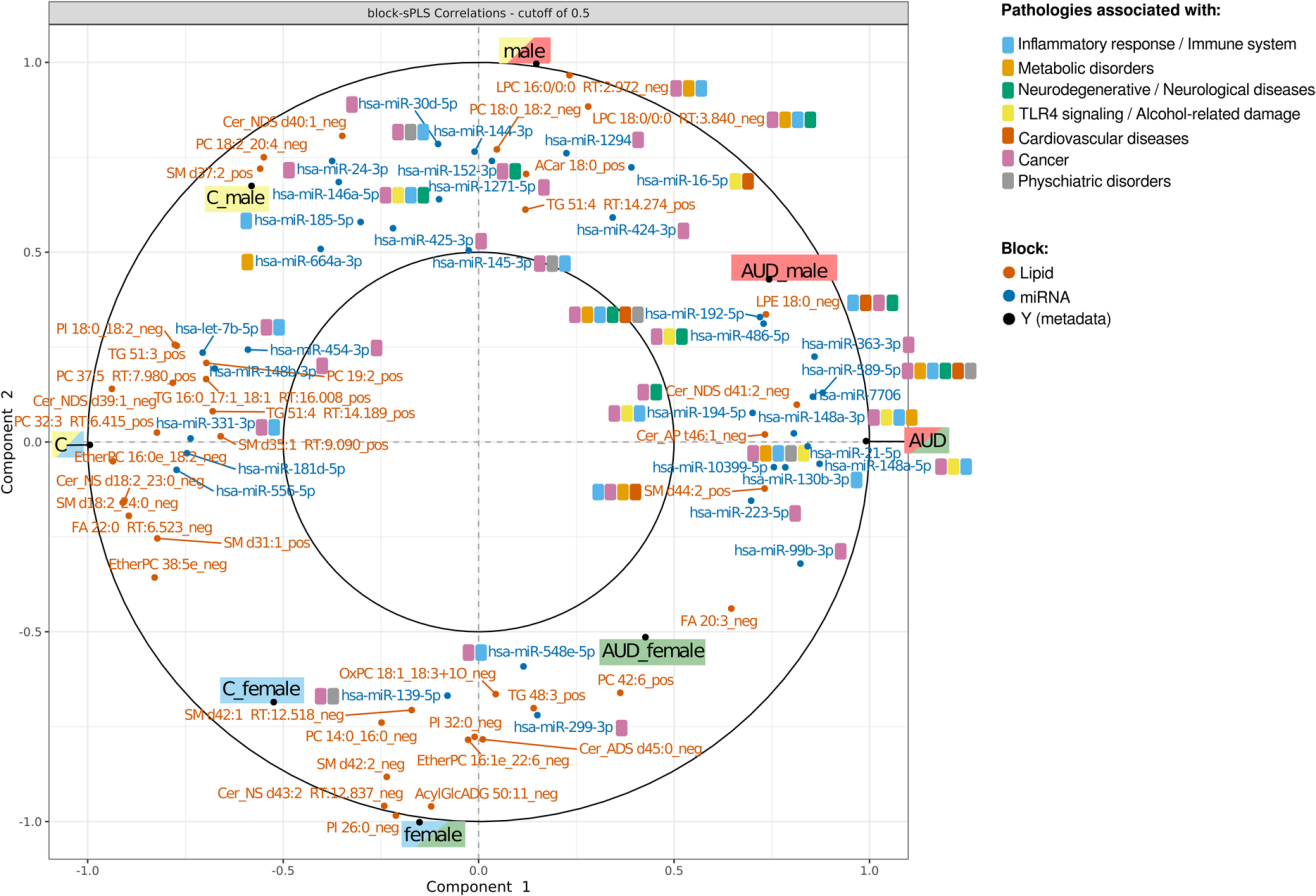
**Varplot from the block.sPLS analysis displaying component 1 vs. component 2 (cutoff = 0.5).** The plot displays the correlations between selected variables from the miRNA and lipidomic blocks and the experimental groups (Y block), projected onto the first two components. Only variables with a correlation ≥|0.5| are shown. Labels are colored by block (lipid, miRNA, or Y), and icons indicate the associated pathologies based on literature annotation. Sample group centroids are highlighted (e.g., AUD female, control male), and distribution patterns reveal distinct molecular signatures by sex and alcohol exposure

A correlation plot with a cutoff of 0.5 illustrated the relationship between the miRNomic and lipidomic datasets and the experimental groups (Fig. 5). In line with our focus on integrating these molecular features into a comprehensive physiological and pathological framework, Fig. 5 highlights those lipids and miRNAs associated with pathologies/terms related to alcohol consumption (Table S6 provides more detailed information). The features positioned in the upper and lower areas associate with males and females, respectively, while the features on the right and left sides correspond to AUD and control patients, respectively.

Table 1 presents the AUD signature of 20 miRNAs and 20 lipids identified through the integrated joint model that maximizes the separation between AUD patients and controls. This multivariate panel effectively captured the combined discriminative power of both molecular layers; additionally, by integrating results from univariate linear regression analyses (DEA: Differential expression analysis of miRNome data, DAA: Differential abundance analysis of lipidome data), we highlighted the miRNAs and lipids that individually differentiate the experimental groups in at least one sex.

**Table 1:** AUD signature based on multi-omics integration (component 1)

Feature	Pattern	DEA/DAA	Feature	Pattern	DEA/DAA
Cer_AP t46:1_neg	+	F	Cer_NS d18:2_23:0_neg	-	F & M
Cer_NDS d41:2_neg	+		EtherPC 16:0e_18:2_neg	-	F & M
FA 20:3_neg	+	M	EtherPC 38:5e_neg	-	M
LPE 18:0_neg	+		FA 22:0 RT:6.523_neg	-	
SM d44:2_pos	+		PC 19:2_pos	-	M
hsa-miR-10399-5p	+	M	PC 32:3 RT:6.415_pos	-	
hsa-miR-130b-3p	+		PC 37:5 RT:7.980_pos	-	
hsa-miR-148a-3p	+	M	PI 18:0_18:2_neg	-	M
hsa-miR-148a-5p	+	M	SM d18:2_24:0_neg	-	F & M
hsa-miR-192-5p	+	M	SM d31:1_pos	-	
hsa-miR-194-5p	+	F	SM d35:1 RT:9.090_pos	-	
hsa-miR-21-5p	+	M	TG 16:0_17:1_18:1 RT:16.008_pos	-	
hsa-miR-223-5p	+		TG 51:3_pos	-	
hsa-miR-340-5p	+	M	TG 51:4 RT:14.189_pos	-	
hsa-miR-363-3p	+	M	hsa-let-7b-5p	-	M
hsa-miR-486-5p	+	M	hsa-miR-148b-3p	-	
hsa-miR-589-5p	+		hsa-miR-181d-5p	-	
hsa-miR-7706	+	M	hsa-miR-331-3p	-	
hsa-miR-99b-3p	+	M	hsa-miR-454-3p	-	
			hsa-miR-556-5p	-	M

This panel includes 40 features (lipids and miRNAs) identified through block.sPLS. Positive (+) or negative (-) symbols indicate the correlation of the feature with the AUD condition. DEA/DAA column indicates the sex where the feature presented a statistical significance in the individual regression analyses

*AUD* Alcohol use disorder, *DEA* Differential Expression Analysis of miRNome, *DAA* Differential Abundance Analysis of lipidome/M: male, F: female

### Divergent patterns based on AUD and sex captured by component 3

**Fig. 6:**
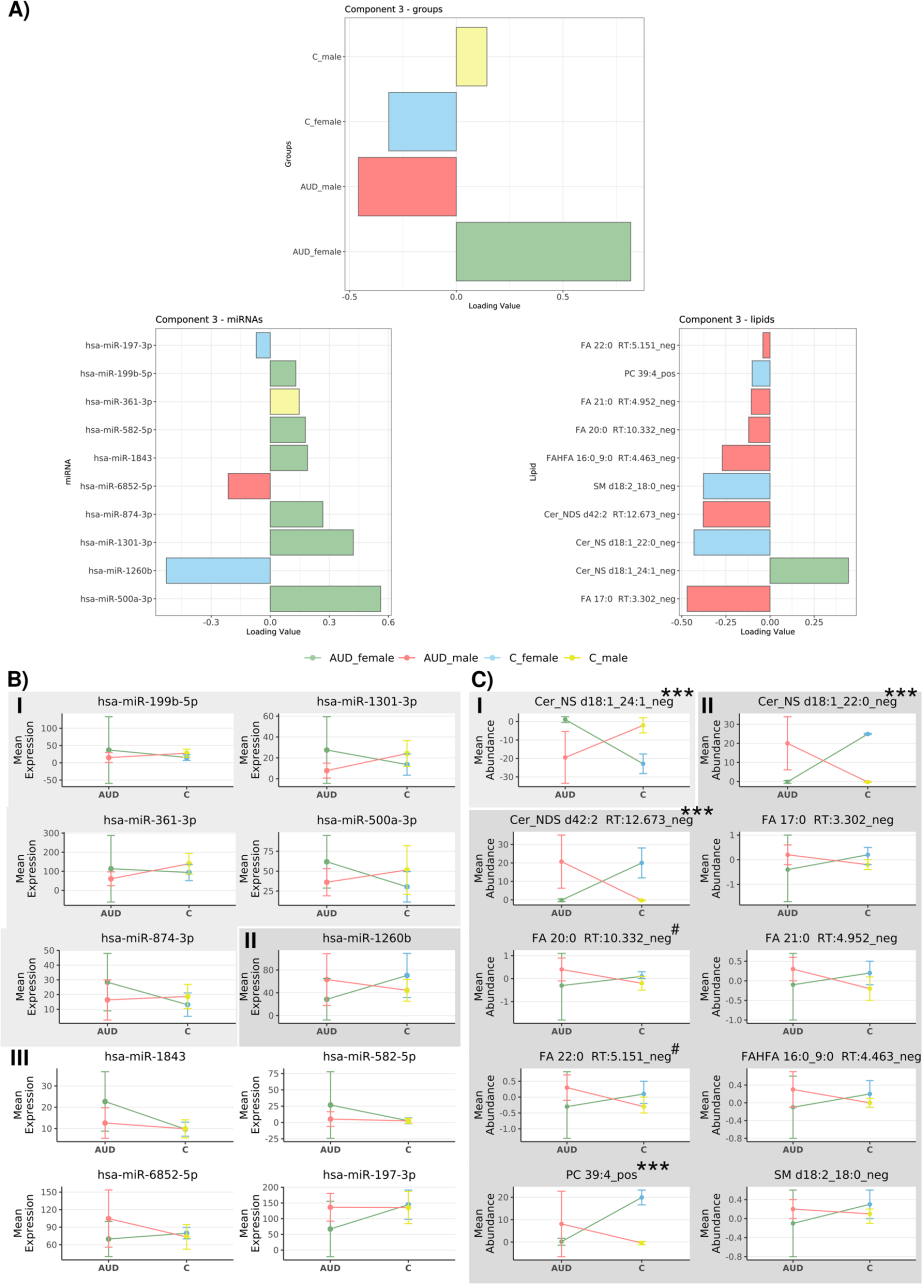
**AUD-sex signature reveals divergent patterns by sex and AUD status.** **A**) Loading plot from block.sPLS integration for Component 3. **B**) Mean expression levels of miRNAs and **C**) mean abundance of lipids that explain the co-variance in Component 3 across experimental groups. # denotes a trend (adjusted p-value < 0.01) and *** denotes statistical significance (adjusted *p*-value < 0.001) in the regression analysis (IS comparison of differential expression/abundance analysis). Pattern I (light grey): higher in AUD_Female and lower in AUD_Male compared to controls. Pattern II (dark grey): lower in AUD_Female and higher in AUD_Male compared to controls. Pattern III (white): controls of both sexes display similar normalized values, while only one sex shows an increase or decrease in AUD. Detailed sample correspondence across omics is provided in Table S1

Figure 6A displays the loading plots for Component 3, highlighting opposing patterns between the main experimental groups and a set of 10 miRNAs and 10 lipids that explain the variance in this component (AUD-sex signature). Figure 6B-C presents the mean values of these variables to visualize miRNA expression and lipid abundance patterns across the experimental groups. Three main patterns emerge from these results: (I) the pattern in light gray indicates an increase in AUD females and a decrease in AUD males compared to controls (e.g., hsa-miR-1301-3p and Cer_NS d18:1_24:1) (Figure S2-A1); (II) the pattern in dark gray indicates a decrease in AUD females and an increase in AUD males relative to controls (e.g., hsa-miR-1260b and Cer_NS d18:1_22:0) (Figure S2-B6); and (III) the pattern in white indicates stability when comparing controls of both sexes, with a specific variation (increase or decrease) in one sex within AUD patients relative to its corresponding control (e.g., hsa-miR-197-3p) (Figure S2-A2, -B7, and B9). Patterns I and II exhibit an apparent cross-over effect, as the response to AUD follows an opposing trend between sexes, with increases in one sex corresponding to decreases in the other. These patterns suggest a sex-dependent response to AUD, where specific miRNAs and lipids exhibit opposite expression trends between males and females. Notably, we found that specific lipids identified in these patterns displayed significance or a trend towards significance in the individual analysis for the IS comparison Perpiñá-Clérigues et al. 2024, providing complementary support for sex-related molecular differences in AUD. In contrast, the transcriptomics analysis did not identify individually significant miRNAs in the IS comparison (Fig. 2); however, a set of miRNAs emerged through the integrative approach, highlighting the influence of sex in AUD. Table 2 summarizes the AUD-sex signature.

**Table 2:** AUD-sex signature based on multi-omics integration (component 3)

Feature	Pattern	DEA	Feature	Pattern	DAA
hsa-miR-1301-3p	I		Cer_NS d18:1_24:1_neg	I	x
hsa-miR-199b-5p	I		Cer_NS d18:1_22:0_neg	II	x
hsa-miR-361-3p	I		Cer_NDS d42:2 RT:12.673_neg	II	x
hsa-miR-500a-3p	I		FA 17:0 RT:3.302_neg	II	
hsa-miR-874-3p	I		FA 20:0 RT:10.332_neg	II	#
hsa-miR-1260b	II		FA 21:0 RT:4.952_neg	II	
hsa-miR-1843	III		FA 22:0 RT:5.151_neg	II	#
hsa-miR-197-3p	III		FAHFA 16:0_9:0 RT:4.463_neg	II	
hsa-miR-582-5p	III		PC 39:4_ pos	II	x
hsa-miR-6852-5p	III		SM d18:2_18:0_neg	II	
I	AUD_Female, C_Male				
II	AUD_Male, C_Female				
III	One sex remains stable				

This panel includes 20 features (lipids and miRNAs) identified through block.sPLS. The pattern indicates the group where the feature is higher in each sex (AUD or Control group). Pattern I: higher in AUD_Female and lower in AUD_Male compared to controls. Pattern II: lower in AUD_Female and higher in AUD_Male compared to controls. Pattern III: controls of both sexes display similar normalized values, while only one sex shows an increase or decrease in AUD. X in the DEA and DAA columns indicates statistical significance (adjusted p-value < 0.01) in the IS comparison in the individual regression analyses (DEA/DAA); # denotes a trend towards significance (0.05 < adjusted p-value < 0.055). *AUD* Alcohol Use Disorder, *DEA* Differential Expression Analysis of miRNome, *DAA* Differential Abundance Analysis of lipidome, *F* female, *M* male, *IS* Impact of Sex Comparison

## Discussion

This study characterizes the molecular landscape of AUD through a sequential analytical framework, moving from single-omic differential expression to multi-omic integration of EV-derived miRNAs and lipids. Our miRNA individual analysis first identified sex-specific miRNA dysregulations, which were further validated and contextualized within alcohol-related pathologies. While lipidomic or miRNomic analyses of EVs have been previously applied to AUD research (Perpiñá-Clérigues et al. 2024; Wang et al. 2024), the present work represents the first to implement a multi-omics integration of both layers, providing a more comprehensive molecular perspective of the disorder. To capture the biological complexity that isolated markers may overlook, we implemented a sparse Partial Least Squares (sPLS) model. This integrative approach identified two distinct molecular signatures based on the maximization of covariance: 1) an AUD signature (20 miRNAs and 20 lipids) differentiating AUD individuals from controls, and 2) an AUD-sex signature (10 miRNAs and 10 lipids) highlighting sex-based divergence. By identifying these highly correlated features, our findings establish a robust, hypothesis-generating framework that opens new avenues for understanding the molecular underpinnings of AUD.

Prior to integration, we performed single-omic univariate differential analyses of lipidomic (Perpiñá-Clérigues et al. 2024) and miRNomic datasets to identify individual features associated with AUD and sex. These analyses allowed us to explore potential biomarkers with strong individual effects and characterize group- and sex-specific expression patterns in each distinct molecular layer. In the context of miRNomics, we identified 7 (females) and 16 (males) significantly altered miRNAs, with only one miRNA in common. These overlapping features offer additional robustness, suggesting sex-specific biomarker candidates with potentially greater individual effect sizes (e.g., hsa-miR-99b-3p). The RT-qPCR validation confirmed the dysregulation of six key miRNAs in alcohol use disorder (AUD), revealing sex-specific patterns and pathological implications. hsa-let-7b-5p was downregulated in AUD males, consistent with prior findings linking it to hippocampal neurodegeneration and addiction (Gowen et al. 2021; Wang et al. 2024) while ethanol-induced neuroimmune responses involve let-7b/HMGB1 release from microglia (Coleman and Crews 2018). hsa-miR-194-5p showed upregulation in AUD females, aligning with studies on neuron-derived EVs in heavy drinkers (Yakovlev et al. 2024) and alcoholic brain tissue (Lewohl et al. 2011), and its association with NF-κB signaling suggests roles in inflammation and hepatocarcinoma (Bao et al. 2015). hsa-miR-21-5p was upregulated in AUD males, tied to chronic AUD via IL6R/STAT3-mediated neuroinflammation (Noutsios et al. 2017) and liver disease (Rodrigues et al. 2023), contrasting with acute alcohol exposure studies (Ibáñez et al. 2020). These findings highlight the potential of these miRNAs as sex-specific biomarkers and underscore their roles in AUD-related neurodegeneration, immune activation, and organ damage, emphasizing the need to distinguish acute versus chronic alcohol effects in biomarker research.

AUD exerts complex biological effects, increasing the risk of cardiovascular, neurological, metabolic, hepatic, and oncological diseases (Bagnardi et al. 2015; Obad et al. 2018). Although we possess data regarding sex-based differences in AUD, particularly in inflammatory responses (Flores-Bonilla 2020; Maddern et al. 2024), they are frequently overlooked in research. The historic underrepresentation of females in AUD studies has resulted in critical gaps in understanding, diagnosis, and treatment, which may contribute to poorer clinical outcomes and underscore the urgent need for more inclusive and sex-informed research and therapeutic strategies (Guinle 2020). In our study, we identified sex-specific miRNomic and lipidomic signatures associated with alcohol-induced molecular alterations, including divergent functional responses to ethanol exposure. Several affected pathways related to inflammation, autophagy, and cellular stress, representing processes commonly implicated in alcohol-related pathologies.

Our data also revealed pronounced sex-specific alterations in autophagy-related regulatory pathways, including those mediated by p53, ubiquitination, Transforming Growth Factor beta (TGF-β), TORC1, the MAPK cascade, mTOR, ErbB signaling, and histone H3 methylation. Prior studies underscored the importance of autophagy in responses to substance-induced stress, particularly alcohol exposure (Pla et al. 2016, 2014; Yu et al. 2024) and alcohol-associated pathologies such as hepatotoxicity (Ni et al. 2013). For instance, alcohol triggers p53-mediated apoptosis in neural crest cells by suppressing TORC1 activity and ribosomal biogenesis, illustrating the complex interplay of these pathways in alcohol-induced neurotoxicity (Huang et al. 2024). Chronic alcohol intake also inhibits autophagy and enhances apoptosis in hepatic tissue (Menk et al. 2018); moreover, several reports documented sex-based differences in autophagy regulation (Lista et al. 2011; Oliván et al. 2014), which may help to explain the divergent responses observed in our data in AUD. While derived from plasma extracellular vesicle signals that preclude direct inference of tissue-specific origin, these differences may contribute to differential susceptibility to alcohol-induced organ damage and inform sex-specific therapeutic strategies targeting autophagy. These sex-dependent molecular patterns extended to oxidative stress responses as well. Although evidence suggests that males are generally more susceptible to oxidative damage and chronic inflammation, whereas females—particularly before menopause—tend to exhibit stronger inflammatory responses and enhanced antioxidant capacity (Martínez De Toda et al. 2023), women are generally more susceptible to developing liver disease or cardiomyopathy after consuming alcohol than men, as well as having a higher incidence of depression and anxiety (Mogos et al. 2019). Although direct evidence for sex-specific stress granule responses to chronic alcohol exposure are limited, cytoplasmic stress granule formation has been reported in *Saccharomyces cerevisiae* under ethanol stress (Kato et al. 2011), and sex differences in stress granule composition have been documented in murine models during cold exposure (Cheung et al. 2024). Our data revealed a divergent expression pattern consistent with sex-specific regulation of stress granules, and one miRNA from our AUD–Sex signature, hsa-miR-197-3p, has been linked to exosomal miRNAs in depression (Zhang et al. 2018) and to circulating miRNAs associated with endothelial dysfunction and cardiometabolic risk in conditions such as Kawasaki disease (McManus et al. 2017).

Finally, the sphingomyelin biosynthetic process emerged in our miRNA sex-differential analysis, reinforcing the notion of sex-specific regulation of sphingolipid metabolism in alcohol-induced brain alterations. Previous lipidomic studies on the same individuals highlighted the role of acid sphingomyelinase in modulating emotional phenotypes and its association with sex-related comorbidities, such as depression and anxiety in AUD (Perpiñá-Clérigues et al. 2024). Moreover, a lipid-driven mechanism involving sphingomyelinases and mitochondria-associated endoplasmic reticulum membranes has been implicated in ethanol-induced EV secretion in glial cells (Ibáñez et al. 2021). This finding agrees with previous reports describing sexual dimorphism in white matter vulnerability and behavioral outcomes in AUD patients (Erol and Karpyak 2015). Our current findings support the hypothesis that sex-specific activity in sphingolipid pathways contributes to divergent neurobiological trajectories in males and females following chronic alcohol exposure. Furthermore, recent evidence has revealed that ceramide production via neutral sphingomyelinase 2 regulates exosomal miRNA secretion and that inhibiting this pathway reduces exosome formation (Kosaka et al. 2010; Mittelbrunn et al. 2011), a process that also appears to be modulated by sex.

Multi-omic analysis proved particularly valuable for uncovering sex-related differences in disease mechanisms by capturing complex expression patterns that remained undetected in single-omic univariate comparisons (Erol and Karpyak 2015). In the miRNA differential expression analysis, no individual features reached statistical significance in the IS comparison; however, the integrative multi-omic analysis identified a coordinated set of sex-associated miRNAs, underscoring the influence of sex in AUD when analyzing distinct omic layers together. Notably, Component 3 revealed a distinct AUD–sex signature, suggesting that sex-based differences in AUD may not only arise from differences in magnitude but also from coordinated multivariate expression patterns across omic layers (e.g., upregulation in one sex and stable expression in the other). Within this multivariate context, 6 of the 10 lipids contributing to Component 3 showed statistical significance or consistent trends in the independent lipidomic IS analysis (Perpiñá-Clérigues et al. 2024). These lipids belong to classes previously associated with male sex, inflammation, and hepatotoxicity, including ceramides (Cer), sphingomyelins (SM), and fatty acids (FA). Moreover, a substantial proportion of the 40 lipids contributing to AUD- and sex-based separations in Components 1 and 2 overlapped with the most deregulated species identified in the single-omics analyses, particularly lysophosphatidylcholines (LPC), phosphatidylcholines (PC), sphingomyelins (SM), and ceramides (Cer). This supports the interpretation of sex-related effects as distributed molecular shifts rather than driven by individual strongly significant features.

As emerging disciplines, multi-omics and EV analyses are powerful but still evolving approaches. A key limitation of this study is the relatively small number of plasma samples from AUD females, compounded by the under-recognition and underreporting of alcohol-related problems in women (McCaul et al. 2019). In addition, EV isolation methods may influence vesicle purity and downstream molecular profiles, and the approach used here was constrained by the limited sample volume available (Greenberg et al. 2025; Rai et al. 2025). Multi-omics integration also presents inherent challenges, including partial overlap across omic layers, reduced sample size after matching datasets, and the inability to directly infer causal relationships between molecular layers (Das and Mukhopadhyay 2021). Moreover, the lack of key clinical and metabolic covariates (e.g., body mass index, fasting status, and comorbidities) may introduce unmeasured variability. Finally, validation of the integrative signature is limited by the need for independent cohorts with matched lipidomic and miRNA data from the same individuals, which are currently not available for comparable AUD populations.

In conclusion, this study proposes an AUD-associated molecular signature that may provide a framework for future predictive modelling approaches and hypothesis generation in the context of alcohol use disorder. In addition, the AUD–sex signature captures sex-specific molecular patterns that may contribute to a better understanding of differential biological responses to alcohol exposure between females and males.This aspect also highlights the persistent gap in female representation in alcohol-related research. Together, our findings support the identification of biologically grounded and sex-informed molecular patterns with potential translational relevance, although further validation in independent cohorts is required before clinical application. These results may ultimately inform the development of non-invasive biomarker panels to improve the detection, monitoring, and personalised management of AUD.

## Supplementary Information

Below is the link to the electronic supplementary material.

Supplementary file1 (DOCX 4.66 MB)

## Data Availability

The normalized and analyzed datasets, along with the programming scripts, are available in the GitHub repository https://github.com/carlapercle/SexEVEthOmics and through the interactive web platform https://carpercle.shinyapps.io/SexEVEthOmics/. The small RNA-seq data have been deposited in the NCBI Sequence Read Archive (SRA) under accession number PRJNA1291119.
